# Promoting Healthy Aging: Physical Activity and Its Dual Effects on Physical Health and Cognitive Function in Chinese Older Adults

**DOI:** 10.3389/fpubh.2025.1561060

**Published:** 2025-03-04

**Authors:** Xing Li, Chen Li

**Affiliations:** School of Management, Shanghai University of Engineering Science, Shanghai, China

**Keywords:** physical activity, physical health, cognitive performance, seniors, ordered logit model

## Abstract

**Background:**

With the acceleration of societal aging, the physical health and cognitive function issues of seniors have increasingly garnered widespread attention. This article explores the impact of physical activity on the physical health and cognitive performance of seniors, aiming to provide a theoretical basis for health management and related policy formulation for seniors, which holds significant academic value and social significance.

**Methods:**

This study constructs an ordered logit regression model to analyze the effects of physical activity on the physical health and cognitive performance of seniors, serving as the baseline model, and conducts a parallelism test to verify the model’s applicability. To ensure the robustness of the results, various methods were employed for testing, including model substitution, replacement of independent and dependent variables, and the use of propensity score matching analysis. Through these methods, the marginal effects of physical activity on the physical health and cognitive performance of seniors were revealed, and further analysis was conducted on the heterogeneity of physical health and cognitive performance among different groups and regions of seniors.

**Results:**

(1) Physical activity has a significant promoting effect on the physical health and cognitive performance of seniors. Seniors who engage in physical activity demonstrate markedly better physical health and cognitive abilities compared to those who do not participate in physical activity, indicating that physical activity has a positive effect on improving the physical health and cognitive performance of seniors. (2) Physical activity exhibits notable marginal effects on the physical health and cognitive performance of seniors. The probability of seniors who participate in physical activity experiencing improvements in physical health and cognitive performance significantly increases, while those who do not engage in physical activity show the opposite trend, with a decrease in the probability of improvement. (3) There is significant heterogeneity in the effects of physical activity on the physical health and cognitive performance of different senior groups. Specifically, seniors who are female, married, or living in urban areas exhibit more pronounced improvements in health and cognition after engaging in physical activity, indicating that the benefits of physical activity are particularly prominent in these groups.

**Discussion:**

Seniors who participate in physical activity demonstrate significantly better physical health and cognitive abilities compared to those who do not engage in physical activity, suggesting that physical activity not only helps to delay physical aging but also effectively slows cognitive decline. Future policies should focus on enhancing the promotion and implementation of physical activity among seniors, especially within groups with differentiated needs, to advance the process of healthy aging and further enhance the overall well-being of the senior population.

## 1 Introduction

With the acceleration of global aging, the physical health and cognitive function issues of the older adults population have become a significant challenge in the field of global public health. According to the World Health Organization’s definition, health is not merely the absence of disease or infirmity, but a complete state of physical, mental, and social well-being. Therefore, improving the overall health level of the older adults population, particularly in terms of physical function and cognitive ability, has become an important issue in health policies across countries worldwide. In 2020, the World Health Organization released the “Global Guidelines on Physical Activity for Older Adults,” advocating that regular participation in physical activities helps older adults improve their physical health, enhance muscle strength, and increase balance, while also promoting mental health and cognitive function, thereby reducing the risk of depression and anxiety. China’s “Healthy China 2030 Planning Outline” also clearly states that the health of the older adults is a key component in achieving the Healthy China strategy, emphasizing that physical activity should be an effective means to promote the health of older adults, capable of preventing chronic diseases, delaying physical decline, and improving cognitive function.

According to existing research, the main factors influencing the health status of the older adults can be categorized into five major categories: natural attributes, socio-economic status, lifestyle, health conditions, and psychological factors (1). Among these five factors, lifestyle is a crucial determinant of health throughout the entire life course. Lifestyle generally includes smoking, alcohol consumption, physical activity, and participation in various activities. Among these, physical activity has become an internationally recognized strategy for promoting health in middle-aged and older adults due to its low cost and ease of participation (2). However, the proportion of older adults individuals participating in physical activities is currently low in many countries around the world, with a preference for moderate to low-intensity activities. Moreover, a common trend is that the level of participation in physical activities gradually declines with age (3–8). Therefore, there is an urgent need to encourage more older adults individuals to engage in physical activities, promote the implementation of healthy aging, and overall enhance the physical and mental health levels of the older adults population.

Currently, research on physical activity and the physical health and cognitive performance of seniors mainly focuses on the following aspects.

Firstly, the duration, intensity, and type of physical activity have an impact on the physical health and cognitive performance of the older adults. The benefits of physical activity for the physical health and cognitive performance of older adults are undeniable (1, 9). Existing research has found that physical activity is significantly positively correlated with both subjective and objective health; the higher the frequency of participation in physical activities, the better the subjective and objective health status (3). However, the “frequency” of physical activity does not adequately explain the “duration, intensity, and type,” which requires further investigation. Chen classified physical activity intensity into vigorous, moderate, and light levels, and the propensity score matching results showed that participating in moderate activities could increase the probability of healthy aging for seniors by 0.76–0.78%, while vigorous and light physical activities had no significant impact on healthy aging (10). The research by Uchida indicated that low-intensity physical activity could improve lung function and physical condition, and is recommended for promoting the health of seniors (11). Other studies found that physical-cognitive dual-task physical activity games had the best intervention effects, followed by physical-cognitive combined activity games, and lastly, single physical or cognitive training. The frequency of physical activity game interventions was generally three times per week, with a duration primarily of 12 weeks, and intervention times ranging from 18 to 90 min, with each session gradually increasing from less to more, ultimately reaching 30 or 50 min (12–14). Carta verified through randomized controlled trials the effectiveness of moderate-intensity physical activity on the cognitive performance of seniors, indicating that moderate-intensity exercise could improve seniors’ cognitive performance, particularly in memory and visual–spatial skills (15).

Secondly, the impact and mechanisms of physical activity on the physical health and cognitive performance of seniors. Physical activity can enhance the physical, mental, and social functions of the research subjects, improve the physical condition of seniors, and increase their immune system strength. For seniors with certain chronic diseases, it helps alleviate or maintain their conditions, thereby improving their physical condition and promoting physiological health. Svobodová et al. found that physical activity could improve seniors’ aerobic capacity and endurance, with seniors engaging in physical activity showing significant improvements in indicators such as the 10-meter walking test and the 6-min walking test, indicating that endurance training helps enhance seniors’ muscle strength and endurance, as well as improve coordination and balance, thus enhancing walking ability (16). Fang Guoliang et al. found that high-intensity interval training could significantly improve seniors’ short-term memory, reaction speed, and resistance to interference in cognitive functions, although its effect on improving attention was not significant (17). Although there is no direct research indicating specific mechanisms through which physical activity affects seniors’ cognitive functions, physical activity can improve cardiovascular function, increase blood supply and oxygen delivery to the brain, and sufficient blood and oxygen supply can maintain normal metabolism and function of neurons, enhancing the synthesis and transmission efficiency of neurotransmitters, thereby contributing to improved cognitive abilities.

Thirdly, there are differences in the impact of physical activity on the physical health and cognitive performance of older adults individuals with different characteristics. Among those who engage in regular physical activity, the probability prediction values for both subjective and objective health for middle-aged and older men are higher than those for middle-aged and older women. Regular physical activity has a greater positive effect on the health levels of middle-aged and older men who have experienced famine. Additionally, urban older adults individuals who have experienced famine and participate in physical activity occasionally or regularly have higher health probability prediction values than their rural counterparts with the same physical activity habits and famine experience (3). Research by Gonçalves A K and others found that physical fitness improves with training, but the effects vary across different age groups: physical training has a positive impact on the 60–69 and 70–79 age groups, while it has no significant effect on those aged 80 and above (18).

It is evident that the consensus in academia is that physical activity can enhance the physical health and cognitive abilities of the older adults, although the focus varies slightly by country. Some studies in European countries pay more attention to the effects of physical activity on brain structure and neural plasticity, while research in developing countries tends to emphasize the prevention and control of common chronic diseases through physical activity. Some findings are particularly novel and thought-provoking; for example, in Indonesia, physical activity can increase bone density, reduce fat accumulation, improve body mass index, lower the risk of musculoskeletal system damage, influence dopamine levels and changes in neurotrophic factors, and suppress cognitive decline and dementia. In Malaysia, physical activity is associated with cognitive function in the older adults, with walking improving cognitive abilities. In developed countries, physical activity is considered essential for maintaining physical function in older adults, improving quality of life, and preventing chronic diseases, with dance potentially having the greatest impact on enhancing cognitive abilities in healthy older adults. In China, physical exercise has been shown to significantly improve cognitive function in sedentary older adults patients with diabetes (19–26). However, there are still disagreements in academia regarding the intensity of physical activity, and research on the differences in the impact of physical activity on the physical health and cognitive performance of older adults individuals with different characteristics remains incomplete. Currently, there is no evidence to suggest that longer durations of physical activity, more frequent activities, or higher intensity levels lead to greater functional improvements (27). The optimal intensity of physical activity for enhancing the physical health and cognitive performance of the older adults still needs to be explored. Questions remain, such as whether higher intensity physical activity can lead to better health outcomes for older adults, whether there are marginal effects of physical activity on older adults health, and whether the impact of physical activity varies among different age groups, genders, and regions. There is currently no comprehensive and unified perspective in academia on these issues.

In light of the above issues, this paper aims to verify the impact of physical activity on the physical health and cognitive performance of seniors by constructing an ologit model. Based on this, it will explore the marginal effects of physical activity on seniors with different levels of physical health and cognitive performance, the differences in the effects of light, moderate, and vigorous physical activity on seniors of different age groups, as well as the heterogeneity in terms of gender, marital status, and urban–rural differences. Finally, combined with empirical findings, this study attempts to provide a scientific basis for health management and policy formulation for seniors, thereby promoting the overall improvement of societal health levels. This research not only has theoretical significance but also provides practical guidance for addressing public health challenges in an aging society.

Compared to existing research, the potential marginal contributions of this paper are as follows. First, this paper further verifies the positive impact of physical activity on the physical health and cognitive performance of seniors based on the ologit model, with robust results. Second, it explores the marginal effects of physical activity on seniors with different levels of physical health and cognitive performance. Third, it investigates the differences in the effects of light, moderate, and vigorous physical activity on seniors of different age groups, as well as the heterogeneity in terms of gender, marital status, and urban–rural differences, thereby supplementing existing evidence.

## 2 Data and methods

### 2.1 Data sources and variable selection

#### 2.1.1 Data sources

The data for this study comes from the China Health and Retirement Longitudinal Study (CHARLS). CHARLS is a large-scale household survey led by the National School of Development at Peking University, which is nationally representative and covers 150 counties and 450 villages across 28 provinces (autonomous regions and municipalities) in China. In terms of survey content, the CHARLS questionnaire includes a wealth of individual and family information about middle-aged and seniors, encompassing their physical and mental health status represented by chronic diseases, disability levels, depression, and cognitive abilities, as well as demographic variables such as gender, age, marital status, and education level. This information provides data support for exploring the causal relationship between physical activity and the physical health and cognitive performance of seniors. Therefore, based on the research objectives, this study utilizes the latest fifth wave (2020) national follow-up data, which was officially released to the public on November 16, 2023. After data cleaning, samples of seniors aged 60 and above were retained, resulting in a final valid sample of 11,473.

#### 2.1.2 Variable selection

##### 2.1.2.1 Dependent variables

This article uses physical health and cognitive performance to reflect the health level of seniors, constructing the following indicators based on these two metrics:

First, physical health is composed of 12 categorical variables, including whether the individual has hypertension, diabetes, cancer, lung disease, heart disease, has had a stroke, arthritis, abnormal blood lipids, liver disease, kidney disease, stomach disease, or asthma. This article distinguishes the health status of the older adults based on the number of illnesses using Hu’s method (28). Using the recode command in Stata software, physical health is categorized into five levels: very poor health, poor health, average health, good health, and very good health.

Second, cognitive performance indicators are constructed from two aspects: situational memory and mental memory. Situational memory is represented by phrase memory, which consists of immediate phrase recall and delayed phrase recall. Mental memory is represented by cognitive completeness, which includes date recognition, calculation, and drawing ability. The former is scored from 0 to 10, with higher scores being better, while the latter is scored from 0 to 11, also with higher scores being better. The combined cognitive performance score ranges from 0 to 21, with higher scores indicating better performance. This is based on Hu’s analysis of cognitive function in the older adults from the CHARLS database (29). Using the recode command, cognitive performance is categorized into five levels: very poor, poor, average, good, and very good.

##### 2.1.2.2 Core explanatory variable

The core explanatory variable is a binary variable indicating whether seniors aged 60 and above participate in physical activities, where 0 represents non-participation and 1 represents participation.

##### 2.1.2.3 Control variables

Control variables include gender, age, age squared, marital status, education level, the logarithm of family income, the logarithm of per capita family consumption, and the logarithm of financial support from children to parents.

##### 2.1.2.4 Other variables

For alternative variables, ADL and IADL are selected as substitutes for the dependent variable of physical health, while situational memory and mental memory are used as substitutes for cognitive performance. Light physical activities (primarily referring to walking, including moving from one place to another while working or at home, as well as other walking for leisure, exercise, or entertainment), moderate-intensity physical activities (activities that make the respondent breathe faster than usual, such as carrying light objects, cycling at a regular pace, mopping, practicing Tai Chi, or brisk walking), and high-intensity physical activities (intense activities that cause the respondent to breathe heavily, such as carrying heavy objects, digging, farming, aerobic exercise, fast cycling, or cycling with cargo) are selected as alternative variables for the core explanatory variable. For heterogeneity analysis, urban–rural status (0 indicates living in a rural area, 1 indicates living in an urban area), gender (0 indicates female, 1 indicates male), and marital status (0 indicates unmarried, 1 indicates married) are chosen as covariates for the heterogeneity analysis.

#### 2.1.3 Descriptive statistics of the data

Table 1 shows the descriptive statistics of the variables.

**Table 1:** Descriptive statistics of variables.

Variables	*N*	Mean	sd	Min	Max
phyh	11,473	2.900	1.295	1	5
cog	7,142	3.219	1.173	1	5
ac	11,450	0.861	0.346	0	1
lightac	11,449	0.752	0.432	0	1
moderac	11,450	0.497	0.500	0	1
intenac	11,450	0.305	0.460	0	1
gender	11,473	0.482	0.500	0	1
age	11,473	70.046	7.201	60	120
agesqr	11,473	4,958.238	1,056.908	3,600	14,400
marry	11,473	0.774	0.418	0	1
edu	11,473	1.865	1.057	1	4
lninc	10,120	7.001	4.312	0	14.982
lnfacom	8,318	9.391	0.929	0	13.116
lnfinasu	11,473	7.087	3.026	0	13.065
urban	11,473	0.598	0.490	0	1
ADL	11,448	4.422	1.077	1	5
IADL	11,447	4.384	1.130	1	5
memory	9,526	3.166	1.239	1	5
mentim	7,239	3.166	1.123	1	5

In terms of the dependent variables, the average scores for physical health and cognitive performance are 2.90 and 3.22, respectively, indicating that the average score for cognitive performance is slightly higher than that for physical health. Based on the values assigned to physical health and cognitive performance, the health status of the surveyed seniors is at a moderate level. The standard deviation for physical health is 1.30, while the standard deviation for cognitive performance is 1.17, suggesting that the variability in physical health is greater. Both variables have a range of 4, indicating significant health disparities within the old people group.

Regarding different levels of physical activity, the proportions of surveyed seniors who have participated in light physical activity, moderate physical activity, and intense physical activity are 75.20, 49.70, and 30.50%, respectively, indicating that as the intensity of exercise increases, the participation rate among seniors decreases.

In terms of control variables, males constitute 48.20% of the surveyed seniors, indicating a relatively balanced gender distribution. The average age of the respondents is 70.05 years, with a minimum age of 60 years and a maximum age of 120 years. Regarding marital status, the proportion of those who are married reaches 70.05%, while nearly 30% of the senior respondents are not married, including widowed, unmarried, and divorced individuals.

In terms of educational attainment, 52.29% of the respondents have an education level of elementary school or below, 20.23% have completed elementary school, 16.20% have completed junior high school, and 11.28% have completed high school or higher, indicating that the overall educational level of the respondents is not high. The means for the logarithm of household income, the logarithm of household consumption per capita, and the logarithm of children’s financial support for parents are 7.00, 9.39, and 7.09, respectively.

Regarding other variables, 51.00% of the surveyed seniors live in urban areas, while 49.00% reside in rural areas, indicating a relatively balanced urban–rural distribution among the respondents. Looking at activities of daily living (ADL) and instrumental activities of daily living (IADL), the mean scores after reverse coding are 4.42 and 4.38, respectively, indicating that most seniors do not require assistance with ADL and IADL and can generally manage their daily lives independently. In terms of contextual memory and mental imagery, both have a mean score of 3.17, suggesting that the cognitive performance of seniors is slightly above average.

### 2.2 Research methods

The dependent variables of seniors’ physical health and cognitive performance are classified as ordinal variables, and an ologit regression can be used to estimate the impact of physical activity on seniors’ physical health and cognitive performance. The equation of the ologit model can be expressed as:


(1) $$\log\left[\frac{p_{\left(y\leq 1\right)}}{1-p_{\left(y\leq 1\right)}}\right]=\tau_{1}-b_{1}x_{i}$$

(2) $$\log\left[\frac{p_{\left(y\leq 2\right)}}{1-p_{\left(y\leq 2\right)}}\right]=\tau_{2}-b_{2}x_{i}$$

⋮


(3) $$\log\left[\frac{p_{\left(y\leq j-1\right)}}{1-p_{\left(y\leq j-1\right)}}\right]=\tau_{j-1}-b_{j-1}x_{i}$$

(4) $$\log\left[\frac{p_{\left(y\leq j\right)}}{1-p_{\left(y\leq j\right)}}\right]=\tau_{j}-b_{j}x_{i}$$

In Equations 1–4, $p_{\left(y\leq j\right)}$ represents the cumulative probability of the dependent variable being less than or equal to option j, while the denominator $1-p_{\left(y\leq j\right)}$represents the probability of being greater than option j. The expression $\log\left[\frac{p_{\left(y\leq j\right)}}{1-p_{\left(y\leq j\right)}}\right]$ is the logit of the cumulative probability. On the right side of the equation, there is no constant term a*_j_*; instead, it is replaced by cutting points $\tau_{j}$. $b_{j}$ represents the regression coefficients of the independent variables. From the above equation, it can be seen that the ologit model is not a single model, but rather j models; the ologit model actually estimates *j*-1 effective models (where *j* is the number of ordered categories of the dependent variable).

## 3 Results and analysis

### 3.1 Benchmark regression and diagnostic testing

#### 3.1.1 Benchmark regression

The ologit model uses the maximum likelihood estimation method for parameter estimation, and the interpretation of its regression results is similar to that of the binary logistic model. We will present the odds ratios from the ologit model and explain the baseline models for the dependent variables of physical health and cognitive performance. Models (1) and (2) represent the baseline regression models for physical health, while Models (3) and (4) represent the baseline regression models for cognitive performance.

After four iterations and final convergence, Models (1), (2), (3), and (4) all passed the significance tests (Table 2).

**Table 2:** Ologit model estimating the health influencing factors for seniors.

Var	Model (1)	Model (2)	Model (3)	Model (4)
	phyh	phyh	cog	cog
ac	0.205^***^	0.280^***^	0.554^***^	0.325^***^
	(0.048)	(0.061)	(0.076)	(0.092)
gender		0.330^***^		0.053
		(0.043)		(0.054)
age		−0.323^***^		0.384^***^
		(0.046)		(0.080)
agesqr		0.002^***^		−0.003^***^
		(0.000)		(0.001)
marry		−0.036		0.344^***^
		(0.054)		(0.073)
edu		−0.035		0.741^***^
		(0.021)		(0.027)
lninc		0.007		−0.016^*^
		(0.005)		(0.006)
lnfacom		−0.147^***^		0.200^***^
		(0.023)		(0.031)
lnfinasu		−0.010		0.003
		(0.007)		(0.009)
cut1	−1.514^***^	−14.843^***^	−1.972^***^	14.025^***^
	(0.049)	(1.698)	(0.080)	(2.871)
cut2	−0.061	−13.361^***^	−0.441^***^	15.692^***^
	(0.046)	(1.697)	(0.073)	(2.873)
cut3	0.793^***^	−12.496^***^	0.863^***^	17.270^***^
	(0.046)	(1.696)	(0.074)	(2.875)
cut4	1.977^***^	−11.322^***^	2.131^***^	18.717^***^
	(0.050)	(1.695)	(0.077)	(2.876)
*N*	11,450	7,758	7,142	5,073
pseudo *R*^2^	0.000	0.008	0.002	0.077

Standard errors in parentheses.

^*^p < 0.05, ^***^p < 0.001.

Model (1) shows that the coefficient of the core explanatory variable, physical activity, is statistically significant, with a *p*-value less than 0.05, indicating that there are significant differences in the physical health of seniors. Specifically, seniors who participate in physical activities have noticeably better physical health compared to those who do not. Model (2) indicates that when controlling for variables such as gender, age, agesqr, education, lninc, lnfacom, and lnfinasu, the significance level of the core explanatory variable, physical activity, remains unchanged, with a *p*-value still less than 0.05, further confirming the significant differences in the physical health of seniors. The odds ratio (OR) of the core explanatory variable is 0.205, which is a positive number, reflecting that seniors who participate in physical activities are more likely to have a higher ranking in the dependent variable and better physical health compared to those who do not participate in physical activities (the reference group). In other words, seniors who engage in physical activities are healthier than those who do not. Controlling for other variables, the OR of physical activity is 0.280, indicating that, while keeping other variables constant, seniors who participate in physical activities are increasingly likely to have a higher ranking in the dependent variable. Model (2) further reveals that seniors who engage in physical activities are healthier than those who do not.

Model (3) shows that the coefficient of the core explanatory variable, physical activity, is statistically significant, with a *p*-value less than 0.05, indicating that there are significant differences in the cognitive performance of seniors. Specifically, seniors who participate in physical activities exhibit noticeably better cognitive performance compared to those who do not. Model (4) indicates that when controlling for variables such as gender, age, agesqr, education, lninc, lnfacom, and lnfinasu, the significance level of the core explanatory variable, physical activity, remains unchanged, with a *p*-value still less than 0.05, further confirming the significant differences in the cognitive performance of seniors. The OR of the core explanatory variable is 0.554, which is a positive number, reflecting that seniors who participate in physical activities are more likely to have a higher ranking in the dependent variable and stronger cognitive abilities compared to those who do not participate in physical activities (the reference group). In other words, seniors who engage in physical activities demonstrate better cognitive performance than those who do not. Controlling for other variables, the OR of physical activity is 0.325, indicating that while the likelihood of seniors who participate in physical activities having a higher ranking in the dependent variable has somewhat diminished, Model (4) reveals that seniors who engage in physical activities exhibit stronger cognitive performance compared to those who do not.

#### 3.1.2 Parallelism test

The baseline regression model explains two facts: that engaging in physical activities offers more benefits for seniors, at least in terms of meeting health requirements and enhancing cognitive performance. However, due to the assumption in the ologit model that the proportional odds hold, meaning the effect of the independent variable on the dependent variable remains constant across the categories of the dependent variable, it is necessary to conduct a score test for the proportional odds assumption. This test examines whether the impact of different values of the independent variable on the dependent variable is consistent across the various regression equations. The null hypothesis of the score test for the proportional odds assumption is that the model satisfies parallelism. If the *p*-value is greater than 0.05, it indicates that the model accepts the null hypothesis, thus meeting the parallelism test; conversely, if the p-value is less than or equal to 0.05, the null hypothesis is rejected, indicating that the model does not satisfy the parallelism test. Since parallelism is a prerequisite for using the ologit model, if it cannot be satisfied, it is preferable to use the mlogit model.

After running the ologit regression model, execute the brant command to output the results of the parallelism test for the ologit model. The parallelism test consists of two parts: one part tests the overall model, represented by the row corresponding to “All,” which checks whether all variables together violate the parallelism assumption, while the other part assesses whether the individual effects of each independent variable violate the parallelism assumption.

As indicated in Table 3, regarding the baseline regression model for physical health, both the overall model test and the tests for each independent variable yield relatively small Chi2 values, and the parallelism tests for all variables, including ac, gender, age, agesqr, edu, lninc, lnfacom, and lnfinasu, are not significant, which means that the baseline regression model for physical health fully satisfies the parallelism assumption. In the case of the baseline regression model for cognitive performance, the Chi2 value for the overall model test is relatively large, and the overall model test is significant, indicating that the baseline regression model for cognitive performance does not satisfy the parallelism assumption. Therefore, since the cognitive performance baseline regression model violates the parallelism assumption, how should we address this issue? Given the complexity of social phenomena, it is not uncommon for the ologit model to be used for statistical estimation of qualitative variables in quantitative social science research, even when the parallelism assumption is violated. If it is found that the model violates the parallelism assumption after testing, different measures should be taken based on the specific situation. If the variable that violates the assumption is not a core explanatory variable in the study but rather a control variable, it may not be necessary to be overly concerned about this issue, as long as the effect of the core explanatory variable does not violate the parallelism assumption, indicating that its estimates for the dependent variable are reliable (30). However, if the core explanatory variable violates the parallelism assumption, the ologit model cannot be used for estimation; in this case, the mlogit model can be chosen for estimation. In Table 3, the Chi2 value for the core explanatory variable ac is relatively small, and its parallelism test is not significant, indicating that the core explanatory variable does not violate the parallelism assumption, and the baseline regression model for cognitive performance still possesses explanatory power.

**Table 3:** Brant test of parallel regression assumption.

	Phyh			Cog		
	Chi2	p > chi2	df	Chi2	p > chi2	df
All	29.48	0.338	27	74.00	0.000	27
ac	3.06	0.382	3	3.52	0.318	3
gender	2.73	0.435	3	23.52	0.000	3
age	5.94	0.115	3	12.09	0.007	3
agesqr	5.77	0.124	3	11.10	0.011	3
marry	1.49	0.685	3	1.51	0.680	3
edu	1.53	0.675	3	15.44	0.001	3
lninc	4.83	0.185	3	0.74	0.864	3
lnfacom	2.92	0.405	3	0.85	0.837	3
lnfinasu	0.12	0.989	3	0.35	0.950	3

### 3.2 Marginal effects analysis

Based on the baseline regression and parallelism test, in order to make the analysis results more quantifiable and precise, we run the margins command to measure the health marginal effects of participation in sports activities among different categories of seniors.

First, we examine the marginal effects of participation in sports activities on physical health among the old people (Table 4). We select the probabilities of having very poor physical health (score of 1), poor physical health (score of 2), average physical health (score of 3), good physical health (score of 4), and very good physical health (score of 5) based on participation in sports activities. Holding other variables constant, the probabilities of having very poor physical health for those who do not participate in sports activities and those who do are 19.50 and 15.51%, respectively; the probabilities of having poor physical health are 31.54 and 28.68%; the probabilities of having average physical health are 19.91 and 20.78%; the probabilities of having good physical health are 17.68 and 20.55%; and the probabilities of having very good physical health are 11.37 and 14.48%. This indicates that the probability of having better physical health with participation in sports activities is increasing, while the probability of having better physical health without participation in sports activities is decreasing. The two show a polarized difference, proving that participation in sports activities is beneficial for the physical health of seniors, and the marginal effect on physical health from participating in sports activities is significant.

**Table 4:** Marginal effects of physical activity on the physical health of seniors.

	Margin	Std. err.	*z*	P > |z|	95% conf. Interval	
1#no	0.1950	0.0095	20.61	0.0000	0.1764	0.2135
1#yes	0.1551	0.0042	36.91	0.0000	0.1469	0.1634
2#no	0.3154	0.0073	43.03	0.0000	0.3010	0.3298
2#yes	0.2868	0.0052	55.55	0.0000	0.2766	0.2969
3#no	0.1991	0.0050	39.94	0.0000	0.1894	0.2089
3#yes	0.2078	0.0046	45	0.0000	0.1987	0.2168
4#no	0.1768	0.0069	25.77	0.0000	0.1633	0.1902
4#yes	0.2055	0.0047	43.95	0.0000	0.1964	0.2147
5#no	0.1137	0.0063	18.02	0.0000	0.1014	0.1261
5#yes	0.1448	0.0041	35.1	0.0000	0.1367	0.1528

Second, we examine the marginal effects of participation in sports activities on cognitive performance among the old people (Table 5). We select the probabilities of having very poor cognitive performance (score of 1), poor cognitive performance (score of 2), average cognitive performance (score of 3), good cognitive performance (score of 4), and very good cognitive performance (score of 5) based on participation in sports activities. Holding other variables constant, the probabilities of having very poor cognitive performance for those who do not participate in sports activities and those who do are 9.63 and 7.27%, respectively; the probabilities of having poor cognitive performance are 22.85 and 19.35%; the probabilities of having average cognitive performance are 31.95 and 31.41%; the probabilities of having good cognitive performance are 21.94 and 24.57%; and the probabilities of having very good cognitive performance are 13.62 and 17.41%. This indicates that the probability of having better cognitive performance with participation in sports activities is increasing, while the probability of having better cognitive performance without participation in sports activities is decreasing. The two show a polarized difference, proving that participation in sports activities is beneficial for the cognitive performance of seniors, and the marginal effect on cognitive performance from participating in sports activities is significant.

**Table 5:** Marginal effects of the impact of physical activity on cognitive performance of seniors.

	Margin	Std. err.	*z*	P > |z|	95% conf. Interval	
1#no	0.0963	0.0080	11.98	0.0000	0.0806	0.1121
1#yes	0.0727	0.0036	20.3	0.0000	0.0656	0.0797
2#no	0.2285	0.0109	20.96	0.0000	0.2072	0.2499
2#yes	0.1935	0.0054	35.84	0.0000	0.1829	0.2041
3#no	0.3195	0.0066	48.59	0.0000	0.3067	0.3324
3#yes	0.3141	0.0064	49.13	0.0000	0.3016	0.3266
4#no	0.2194	0.0089	24.53	0.0000	0.2018	0.2369
4#yes	0.2457	0.0059	41.45	0.0000	0.2341	0.2573
5#no	0.1362	0.0101	13.5	0.0000	0.1164	0.1560
5#yes	0.1741	0.0051	34.38	0.0000	0.1642	0.1840

### 3.3 Robustness test

To verify that the results of the benchmark regression model analysis are more convincing, the study conducts robustness tests using methods such as sampling replacement models, replacing independent variables, replacing dependent variables, and propensity score matching analysis.

#### 3.3.1 Replace the model

The ologit model corresponds to the oprobit model; these two models are like twin sisters. They both assume that there is some relationship between the independent variables and the categories of the dependent variable, and they explain the ordinal changes in the dependent variable through the influence of these independent variables. The ologit model models the relationship through linear regression of Log odds, while the oprobit model models the relationship between the dependent and independent variables through a latent normal distribution, and is derived based on the cumulative distribution function. Similarly, four benchmark models are explained for the dependent variable of physical health and the dependent variable of cognitive performance. Models (5) and (6) are the benchmark regression models for physical health, while models (7) and (8) are the benchmark regression models for cognitive performance. The results of the oprobit model (Table 6) show that the coefficient of the core explanatory variable, physical activity, is statistically significant, with a *p*-value less than 0.05, indicating that seniors’ participation in physical activities significantly promotes both physical health and cognitive performance. When controlling for variables such as gender, age, agesqr, edu, lninc, lnfacom, and lnfinasu, the regression coefficient of the core explanatory variable, physical activity, remains significant, suggesting that the replacement model has robust benchmark regression results.

**Table 6:** Oprobit model for estimating factors influencing the health of seniors.

Var	Model (5)	Model (6)	Model (7)	Model (8)
	phyh	phyh	cog	cog
ac	0.121^***^	0.168^***^	0.332^***^	0.205^***^
	(0.028)	(0.036)	(0.044)	(0.054)
		Control Variables		Control Variables
cut1	−0.908^***^	−8.651^***^	−1.121^***^	4.147^**^
	(0.028)	(0.949)	(0.045)	(1.357)
cut2	−0.045	−7.769^***^	−0.281^***^	5.081^***^
	(0.027)	(0.948)	(0.043)	(1.357)
cut3	0.488^***^	−7.232^***^	0.526^***^	6.027^***^
	(0.027)	(0.948)	(0.043)	(1.358)
cut4	1.176^***^	−6.551^***^	1.280^***^	6.871^***^
	(0.029)	(0.948)	(0.045)	(1.358)
*N*	11,450	7,758	7,142	5,073
pseudo *R*^2^	0.001	0.008	0.003	0.077

Standard errors in parentheses.

^**^p < 0.01, ^***^p < 0.001.

#### 3.3.2 Replace the independent variable

Select “whether engaged in light physical activity,” “whether engaged in moderate physical activity,” and “whether engaged in vigorous physical activity” as substitute variables for participation in physical activity, that is, replacing the core explanatory variables. Light physical activity refers to walking, including moving from one place to another while working or at home, as well as other walking done for leisure, exercise, sports, or entertainment, without significant fatigue or shortness of breath, reaching 40–55% of maximum heart rate; moderate physical activity refers to activities of moderate intensity that cause the respondent’s breathing to be slightly faster than usual, such as carrying light objects, cycling at a regular pace, mopping, practicing Tai Chi, or brisk walking, where there is a certain degree of shortness of breath during exercise, but normal conversation is still possible, reaching 55–70% of maximum heart rate; while vigorous physical activity refers to intense activities that cause the respondent to experience shortness of breath, such as lifting heavy objects, digging, farming, aerobic exercise, cycling quickly, or carrying loads while cycling, where there is significant shortness of breath and an increased heart rate during exercise, making it difficult to maintain a coherent conversation for long periods, reaching 70 to 90% of maximum heart rate (31).

It is surprisingly found that different intensities of physical activities have significant differences in their effects on the physical health and cognitive performance of seniors. Light physical activities like walking do not have a significant impact on the physical health of seniors (Table 7), with a positive but insignificant regression coefficient in Model (9). The regression coefficients in Model (10) and Model (11) are positive and significant, indicating that participation in moderate or vigorous physical activities significantly promotes the physical health of seniors, with greater benefits to physical health as the intensity of physical activities increases. Conversely, the regression coefficients in Model (12), Model (13), and Model (14) decline from 0.292 to 0.207 and then to −0.141, indicating that an increase in the intensity of physical activities leads to a decrease in cognitive performance among seniors.

**Table 7:** Robustness test of estimated health influences on seniors: replacing the independent variable.

Var	Model (9)	Model (10)	Model (11)	Model (12)	Model (13)	Model (14)
	phyh	phyh	phyh	cog	cog	cog
lightac	0.088			0.292^***^		
	(0.048)			(0.066)		
moderac		0.164^***^			0.207^***^	
		(0.042)			(0.052)	
intenac			0.303^***^			−0.141^*^
			(0.045)			(0.056)
	Control variables	Control variables	Control variables	Control variables	Control variables	Control variables
cut1	−14.697^***^	−14.763^***^	−14.252^***^	13.947^***^	13.989^***^	13.818^***^
	(1.698)	(1.700)	(1.700)	(2.870)	(2.870)	(2.875)
cut2	−13.218^***^	−13.283^***^	−12.768^***^	15.614^***^	15.656^***^	15.483^***^
	(1.696)	(1.699)	(1.698)	(2.872)	(2.873)	(2.877)
cut3	−12.354^***^	−12.418^***^	−11.900^***^	17.194^***^	17.235^***^	17.060^***^
	(1.696)	(1.698)	(1.697)	(2.874)	(2.875)	(2.879)
cut4	−11.181^***^	−11.244^***^	−10.724^***^	18.643^***^	18.683^***^	18.508^***^
	(1.695)	(1.697)	(1.697)	(2.875)	(2.876)	(2.880)
*N*	7,758	7,758	7,758	5,073	5,073	5,073
pseudo *R*^2^	0.007	0.008	0.009	0.077	0.077	0.077

Standard errors in parentheses.

*p < 0.05, ***p < 0.001.

Why are vigorous physical activities more beneficial for the physical health of seniors, while detrimental to cognitive performance? We attempt a comparative analysis across different age groups, dividing seniors into the 60–69 age group, the 70–79 age group, and those aged 80 and above. The regression results show that as the age group increases, vigorous physical activities are more beneficial for the physical health of seniors. However, in reality, vigorous physical activities may not be suitable for seniors aged 80 and above. Therefore, we further divide seniors aged 80 and above into urban and rural groups. The results reveal that vigorous physical activities are beneficial for the physical health of urban seniors aged 80 and above, with a positive and significant regression coefficient, while the regression coefficient for rural seniors aged 80 and above is not significant. In terms of cognitive performance, vigorous physical activities have a negative impact on the cognitive performance of seniors in the 60–69 age group, while the regression coefficients for cognitive performance in the 70–79 age group and the 80 and above age group are not significant.

#### 3.3.3 Replace the dependent variable

Using ADL and IADL to replace the dependent variable physical health, and using memory and mentim to replace the dependent variable cognitive performance, is highly persuasive. ADL refers to the most basic activities of daily living for individuals, primarily involving the ability for personal self-care. It consists of indicators such as the ability to dress, bathe, eat, use the toilet, walk, and perform activities in bed. IADL refers to relatively complex activities that require higher cognitive and social abilities; these do not involve basic self-care but are crucial for a person to maintain independent living. It includes the ability to cook, clean, shop, manage finances, appear in public, communicate by phone, and manage medication. Both ADL and IADL are important indicators of physical health status. This paper employs the comprehensive scoring analysis method developed by Chen et al. to recode ADL and IADL, where a higher score indicates better physical health (32). Contextual memory is represented through phrase memory, consisting of immediate and delayed phrase recall, while mental image is represented through cognitive completeness, comprising date recognition, calculation, and drawing abilities. Both can reflect cognitive performance.

The regression results of Model (15) and Model (16) show that, while controlling for other variables, seniors’ participation in physical activities has a significant positive effect on ADL and IADL scores, with positive and significant regression coefficients (Table 8). The regression results of Model (17) and Model (18) indicate that, while controlling for other variables, seniors’ participation in physical activities significantly promotes memory and mentim, with positive and significant regression coefficients. These four models validate the robustness of the baseline regression results.

**Table 8:** Robustness test of estimated health influences on seniors: replacing the dependent variable.

Var	Model (15)	Model (16)	Model (17)	Model (18)
	ADL	IADL	memory	mentim
ac	1.126^***^	1.203^***^	0.373^***^	0.272^**^
	(0.069)	(0.069)	(0.074)	(0.092)
	Control variables	Control variables	Control variables	Control variables
cut1	−5.039^*^	0.390	8.619^***^	16.775^***^
	(2.052)	(2.111)	(2.338)	(2.855)
cut2	−4.004	1.693	9.715^***^	18.019^***^
	(2.052)	(2.112)	(2.340)	(2.856)
cut3	−3.480	2.253	11.080^***^	19.361^***^
	(2.052)	(2.113)	(2.341)	(2.858)
cut4	−2.560	2.938	12.786^***^	21.925^***^
	(2.052)	(2.113)	(2.341)	(2.861)
*N*	7,758	7,758	6,585	5,126
pseudo *R*^2^	0.045	0.059	0.066	0.065

Standard errors in parentheses.

*p < 0.05, **p < 0.01, ***p < 0.001.

#### 3.3.4 Propensity score matching analysis

The propensity score refers to the probability of an individual being in any given intervention state (33). This section considers whether to engage in physical activity as a binary intervention variable, with the propensity score representing the probability of this intervention variable taking a value of 1. Propensity score matching analysis has advantages over linear regression, as it can directly obtain estimates of causal effects based on matched samples, while linear regression must include other control variables when estimating causal effects. Common methods for propensity score matching analysis include 1:1 matching, 1:4 matching, radius matching, and kernel matching. For ease of comparison, we present the results of the four matching methods (Table 9). From the 1:1 matching, there is a significant difference in the ATT and ATU values for physical activity regarding phyh, indicating that the health effect for seniors who have engaged in physical activity is 6.21%, while the expected health effect for those who have not engaged in physical activity is 18.89%. The differences in the ATT and ATU values for cognitive performance (cog) are relatively small, with the effect for seniors who have engaged in physical activity being 21.14%, compared to an expected cognitive performance effect of 19.86% for those who have not. From the 1:4 matching, the health effect for seniors who have engaged in physical activity is lower than the expected health effect for those who have not, while the cognitive performance effect is the opposite. From the radius matching, the ATT and ATU values are the same, indicating that the health effects are identical regardless of whether physical activity is undertaken. The results of kernel matching are interpreted similarly to the above. For the 1:1 matching, 1:4 matching, and kernel matching, the health effects from physical activity are lower than those of the untreated group, while the cognitive performance effects are higher than those of the untreated group. This aligns with the lower regression coefficient for phyh and the higher regression coefficient for cog in the baseline regression, indicating that the results of the baseline regression analysis are robust.

**Table 9:** Propensity-matched score analysis of physical activity on physical health and cognitive performance of seniors.

Methods	phyh			cog		
	ATT	ATU	ATE	ATT	ATU	ATE
1:1 matched	0.0621	0.1888	0.0791	0.2114	0.1986	0.2103
1:4 matched	0.1623	0.2168	0.1696	0.2014	0.1945	0.2008
Radius	0.1880	0.1880	0.1880	0.3692	0.3692	0.3692
Kernel	0.1585	0.2080	0.1652	0.2643	0.2660	0.2645

**Figure 1:**
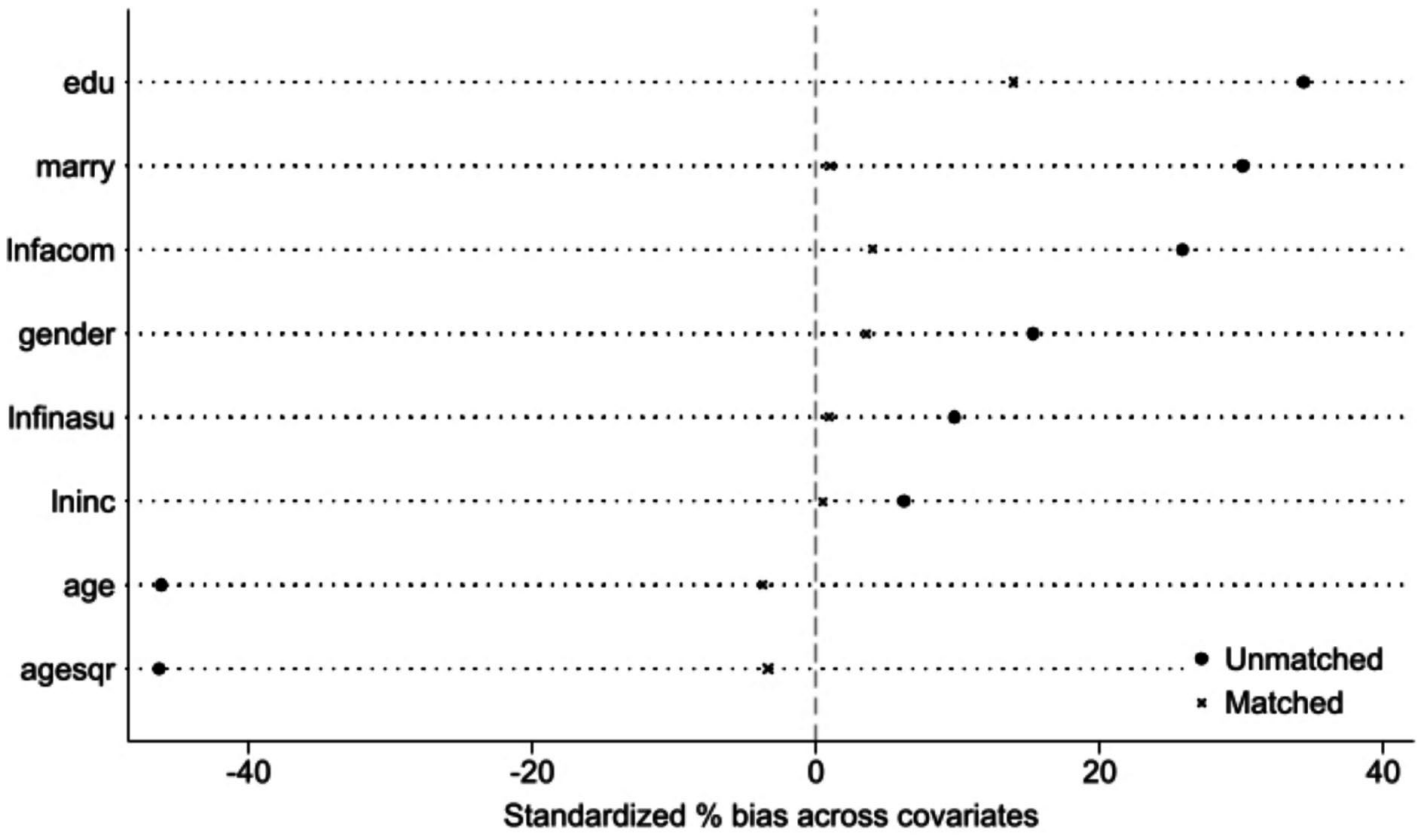
Standardized mean differences of covariates before and after matching.

After completing the propensity score matching analysis, we conducted a balance test on the data using physical activity as an example for the health of the older adults. The kernel propensity score matching method was employed here. The results showed a significant improvement in data balance compared to before matching. Most of the t-test results for the covariates before matching were significant, while the significance levels of the t-tests after matching decreased substantially. The mean and median of the standardized mean differences of the covariates both showed a noticeable decline after matching (Figure 1), reflecting that the B index and R index of overall data balance fell within a reasonable range after matching. The R^2^ after matching also became significant (Table 10), indicating that kernel propensity score matching is robust.

**Table 10:** Propensity score matching balance test.

Sample	Ps R^2^	LR chi2	p > chi2	MeanBias	MedBias	*B*	*R*	%Var
Unmatched	0.054	332.61	0	26.8	28	59.2*	0.62	83
Matched	0.004	67.76	0	3.9	3.5	14.2	1.24	67

^*^if B > 25%, *R* outside [0.2, 2].

### 3.4 Heterogeneity analysis

The heterogeneity of physical activity’s impact on the physical health and cognitive performance of seniors is revealed from three aspects: gender (male and female), marital status (married and unmarried), and urban versus rural settings.

In terms of physical health heterogeneity, physical activity benefits the physical health of both male and female seniors, but in terms of intensity, physical activity is more beneficial for female seniors. Regarding marital status, physical activity benefits the physical health of both married and unmarried seniors, but in terms of intensity, physical activity is more beneficial for married seniors. When examining the physical health status of seniors in urban and rural areas, physical activity benefits the physical health of both urban and rural seniors, but in terms of intensity, physical activity is more beneficial for urban seniors (Table 11).

**Table 11:** Heterogeneity analysis of the ologit model for estimating factors affecting physical health of seniors.

	Model (19)	Model (20)	Model (21)	Model (22)	Model (23)	Model (24)
	Female	Male	Married	Unmarried	Rural	Urban
ac	0.359^***^	0.183^*^	0.482^***^	0.192^**^	0.227^**^	0.409^***^
	(0.083)	(0.091)	(0.113)	(0.073)	(0.074)	(0.109)
	Control variables	Control variables	Control variables	Control variables	Control variables	Control variables
cut1	−15.937^***^	−13.732^***^	−11.721^***^	−15.873^***^	−12.440^***^	−18.321^***^
	(2.362)	(2.394)	(3.115)	(2.276)	(2.135)	(2.751)
cut2	−14.465^***^	−12.236^***^	−10.181^**^	−14.406^***^	−11.016^***^	−16.750^***^
	(2.360)	(2.392)	(3.111)	(2.275)	(2.133)	(2.748)
cut3	−13.620^***^	−11.347^***^	−9.354^**^	−13.529^***^	−10.162^***^	−15.861^***^
	(2.359)	(2.391)	(3.110)	(2.274)	(2.132)	(2.747)
cut4	−12.393^***^	−10.215^***^	−8.168^**^	−12.356^***^	−8.947^***^	−14.752^***^
	(2.358)	(2.391)	(3.109)	(2.273)	(2.132)	(2.745)
*N*	3,924	3,834	1,627	6,131	4,689	3,069
pseudo *R*^2^	0.006	0.007	0.016	0.006	0.006	0.011

Standard errors in parentheses.

*p < 0.05, **p < 0.01, ***p < 0.001.

In terms of the heterogeneity of cognitive performance, physical activity benefits the cognitive performance of both male and female seniors, but in terms of intensity, physical activity is slightly more beneficial for female seniors. Regarding marital status, physical activity benefits the cognitive performance of both married and unmarried seniors, but in terms of intensity, physical activity is more beneficial for married seniors. When examining the cognitive performance of seniors in urban and rural areas, physical activity is beneficial for the cognitive performance of urban seniors, but it does not have a significant impact on the cognitive performance of rural seniors (Table 12).

**Table 12:** Heterogeneity analysis of the ologit model for estimating factors influencing cognitive performance among seniors.

	Model (25)	Model (26)	Model (27)	Model (28)	Model (29)	Model (30)
	Female	Male	Married	Unmarried	Rural	Urban
ac	0.316^*^	0.314^*^	0.535^*^	0.278^**^	0.190	0.514^***^
	(0.136)	(0.126)	(0.216)	(0.102)	(0.115)	(0.155)
	Control variables	Control variables	Control variables	Control variables	Control variables	Control variables
cut1	10.395^*^	16.280^***^	2.728	17.272^***^	7.968^*^	18.864^***^
	(4.440)	(3.815)	(5.793)	(3.484)	(3.772)	(4.419)
cut2	12.044^**^	18.007^***^	4.273	18.984^***^	9.590^*^	20.681^***^
	(4.443)	(3.818)	(5.797)	(3.487)	(3.774)	(4.422)
cut3	13.525^**^	19.675^***^	5.808	20.573^***^	11.222^**^	22.221^***^
	(4.445)	(3.822)	(5.800)	(3.489)	(3.776)	(4.426)
cut4	15.021^***^	21.110^***^	7.371	22.004^***^	12.648^***^	23.721^***^
	(4.445)	(3.824)	(5.803)	(3.490)	(3.777)	(4.429)
*N*	2,170	2,903	811	4,262	2,853	2,220
pseudo *R*^2^	0.103	0.054	0.088	0.071	0.063	0.075

Standard errors in parentheses.

*p < 0.05, **p < 0.01, ***p < 0.001.

## 4 Discussion

This study systematically reveals the mechanism of physical activity on multidimensional health indicators in the older adults population by constructing an ordered logit model and conducting a series of robustness tests. Through marginal effect decomposition and heterogeneity analysis, the research deepens the understanding of intervention pathways and provides new empirical evidence for the design of differentiated health policies. At the foundational mechanism level, the study finds that the promoting effects of physical activity on physical health and cognitive function are statistically significant and biologically plausible. The research reveals that the marginal effects exhibit non-linear characteristics: when the frequency of participation in physical activity exceeds a threshold, health benefits show an accelerating growth trend. Meanwhile, propensity score matching analysis eliminates health selection bias, confirming that the observed health gains primarily stem from the causal effects of physical activity rather than individual self-selection. In terms of heterogeneity analysis, the study identifies significant group differences: female participants, married individuals, and urban residents exhibit stronger intervention responsiveness. This difference may arise from the moderating role of social support networks—emotional support and exercise companionship provided by marital relationships may enhance behavioral compliance; well-equipped fitness facilities and health education in urban communities lower the barriers to participation in physical activity.

This study has three methodological limitations: first, although the classification of health indicators references internationally accepted standards, the setting of classification thresholds may affect the sensitivity of ordered regression parameter estimates; second, insufficient age stratification in the sample may obscure lifecycle effects, necessitating further differentiation of exercise response patterns between younger older adults (ages 65–74) and older adults (ages 75 and above). For instance, the small proportion of the very older adults population (those aged 90 and above only account for 1.2% of the total sample) may not be suitable for high-intensity physical activities, and future research needs to delve deeper to reveal the impact mechanisms of physical activity on older adults health; finally, the absence of mental health variables prevents the study from fully constructing a “physiological-psychological-social” health model. Subsequent research could adopt a mixed-methods design, combining biomarker detection and longitudinal tracking to explore the multidimensional pathways of exercise interventions in depth.

Based on the above findings, it is recommended to optimize healthy aging policies from three levels: first, establish an exercise prescription system based on precision medicine concepts, designing tiered intervention plans for different subgroups; second, improve the age-friendly transformation of community sports facilities, with a focus on enhancing resource allocation in rural areas; third, incorporate exercise adherence assessment into the basic public health service package, building a long-term health promotion mechanism with multi-departmental collaboration. These measures align closely with the life-cycle health management strategy proposed in the “Healthy China 2030 Planning Outline” and hold significant practical value for achieving active aging.

## 5 Conclusion

This article constructs a regression model to analyze the impact of physical activity on the health of seniors, delving into the marginal effects of physical activity on their physical health and cognitive performance, revealing the significant influence of physical activity on senior health. The main conclusions are as follows:

First, physical activity has a significant promoting effect on the physical health and cognitive performance of seniors. There are notable differences in physical health and cognitive performance between seniors who participate in physical activities and those who do not. Specifically, seniors who engage in physical activities outperform their counterparts in both physical health status and cognitive performance. This conclusion has been validated across different model settings, variable substitutions, adjustments of dependent and independent variables, as well as propensity score matching analysis, further demonstrating the robustness of the results obtained using the ologit model.

Second, physical activity exhibits a clear marginal effect on the physical health and cognitive performance of seniors. Participation in physical activities significantly increases the probability of seniors’ physical health status improving positively, whereas seniors who do not engage in physical activities show a declining trend in the probability of positive health developments. Similarly, physical activity has a comparable marginal effect on seniors’ cognitive performance, with those participating in physical activities demonstrating significant improvements in cognitive abilities, while those not participating show no significant enhancement in cognitive abilities. These results indicate that physical activity has a strong marginal effect on both the physical health and cognitive performance of seniors.

Third, the impact of physical activity on the physical health and cognitive performance of different groups of old people shows significant heterogeneity. There are heterogeneous effects of physical activity on the physical health and cognitive performance of various groups of old people. Specifically, the benefits of physical activity are particularly pronounced among female seniors, married seniors, and those living in urban areas, with substantial improvements in their physical health and cognitive performance. In contrast, while physical activity still promotes the physical health and cognitive abilities of male seniors or unmarried seniors, the effects are relatively weaker. This heterogeneity provides a basis for developing more targeted health intervention strategies.

## Data Availability

Publicly available datasets were analyzed in this study. This data can be found here: https://charls.pku.edu.cn/.
